# Human Umbilical Cord Mesenchymal Stem Cell-Derived Exosomes Accelerate Diabetic Wound Healing *via* Ameliorating Oxidative Stress and Promoting Angiogenesis

**DOI:** 10.3389/fbioe.2022.829868

**Published:** 2022-01-31

**Authors:** Chenchen Yan, Yan Xv, Ze Lin, Yori Endo, Hang Xue, Yiqiang Hu, Liangcong Hu, Lang Chen, Faqi Cao, Wu Zhou, Peng Zhang, Guohui Liu

**Affiliations:** ^1^ Department of Orthopedics, Union Hospital, Tongji Medical College, Huazhong University of Science and Technology, Wuhan, China; ^2^ Department of Plastic Surgery, Brigham and Women’s Hospital, Harvard Medical School, Boston, MA, United States; ^3^ Department of Orthopedics, Suzhou Science and Technology Town Hospital, The Affiliated Suzhou Science and Technology Town Hospital of Nanjing Medical University, Suzhou, China

**Keywords:** diabetes, exosome, wound healing, stem cells, endothelial cells

## Abstract

Diabetic wounds remain a great challenge for clinicians due to the multiple bacterial infections and oxidative damage. Exosomes, as an appealing nanodrug delivery system, have been widely applied in the treatment of diabetic wounds. Endovascular cells are important component cells of the vascular wall. Herein, we investigated the effects of HUCMSCs and HUC-Exos (exosomes secreted by HUCMSCs) on diabetic wound healing. In this study, HUVECs were coincubated with HUCMSCs, and HUC-Exos were utilized for *in vitro* and *in vivo* experiments to verify their roles in the regulation of diabetic wound healing. Our results demonstrated that HUCMSCs have the ability to regulate oxidative stress injuries of endothelial cells through exosomes and accelerate diabetic cutaneous wound healing *in vitro*. The present study suggests that HUC-Exos accelerate diabetic cutaneous wound healing, providing a promising therapeutic strategy for chronic diabetic wound repair.

## Introduction

With the diet changes and rising average age of the global population, the prevalence of diabetes is on the rise (Martinengo et al., 2019; Kowluru, 2020). The main comorbidities of diabetes include chronic recalcitrant cutaneous wounds due to multiple drug-resistant bacterial infections, angiopathy, and oxidative damage to the microenvironment (Castleberry et al., 2016; Armstrong et al., 2017). Complications of diabetes are not only leading causes of disability and mortality worldwide but also a significant economic burden to the community (Bowling et al., 2015; Sen, 2019; Chang and Nguyen, 2021). About 22.3 million people in the United States were diagnosed with diabetes. Of which, 15%–25% are at risk for diabetic foot (Reardon et al., 2020). Specifically, up to 2%–3% of diabetics are at risk of developing active diabetic ulcer foot (Lavery et al., 2016). In addition, the recurrence rate of diabetic foot cannot be ignored. According to statistics, 40% of diabetic foot patients have onset symptoms after 1 year, and the probability of recurrence symptoms within 5 years is 65% (Lim et al., 2017). While many therapeutic strategies have been adopted to treat recalcitrant cutaneous wounds, the clinical results for those have been unsatisfactory (An et al., 2021; Deng et al., 2021). In order to solve the problem of refractory wound healing and improve the prognosis, we aim to address the underlying angiopathy by restoring blood supply to the skin and tissue integrity (Wong et al., 2015; Qu et al., 2018; Wang et al., 2019).

Angiogenesis is the basis of blood supply and an important physiological response required to cure diabetic skin wounds. It has been known that vascular endothelial cells are the main driver of angiogenesis (Chen et al., 2018). Previous studies have reported that, under the high-glucose environment caused by diabetes, vascular endothelial cells are damaged by oxidative stress, resulting in low cell activity and decreased proliferation ability (Zhang et al., 2021). For example, the nicotinamide adenine dinucleotide phosphate (NADPH) oxidase (NOX) family is involved in bacterial inhibition, neutrophil chemotaxis, and signal transduction necessary for skin wound healing (Shinohara et al., 2007). Among them, NOX1 and NOX4 play a role in regulating the proliferation and differentiation of HUVECs. In addition, oxygen is essential for wound healing as the wound healing process relies on adenosine triphosphate (ATP) for energy (Thi et al., 2020). Nevertheless, oxygen makes contributions to the expression of other reactive oxygen species (ROS) on the condition that it was transformed into superoxide. Previous studies have shown that NOX activation and ROS release are both related to skin wound healing (Sadjadi et al., 2019).

HUCMSCs are high-profile due to their pluripotency. In recent years of research, HUCMSCs have been applied in many clinical fields, such as *in vitro* organ culture and heart and lung injury repair, made possible due to characteristics such as easier separation, purification, and culture (Pavlou et al., 2018). Some studies have shown that HUCMSCs can promote the formation of new blood vessels and strengthen tissue regeneration (Yin et al., 2021). In the research focusing on the mechanism of HUCMSCs, it was found that the exosomes derived from them have good stability and immunogenicity, with the ability to transport proteins and growth factors of different functions to exert different effects (Zhuang et al., 2020). Recent studies have shown that exosomes derived from HUCMSCs regulate the differentiation and proliferation of bone marrow mesenchymal stem cells (Wen et al., 2020). Studies have also shown that exosomes derived from HUCMSCs can regulate oxidative stress and inhibit cell hypoxia damage (Xue et al., 2018). Based on these findings, exosomes may serve as a promising candidate to promote angiogenesis in wound healing.

This study is thus intended to verify that exosomes from HUCMSCs can enhance angiogenesis by endothelial cells and to determine the effects of HUC-Exos (exosomes secreted by HUCMSCs) on cutaneous wound healing *in vivo*.

## Results

### Hyperglycemia drives oxidative stress damage and impairs cellular function in HUVECs

**FIGURE 1:**
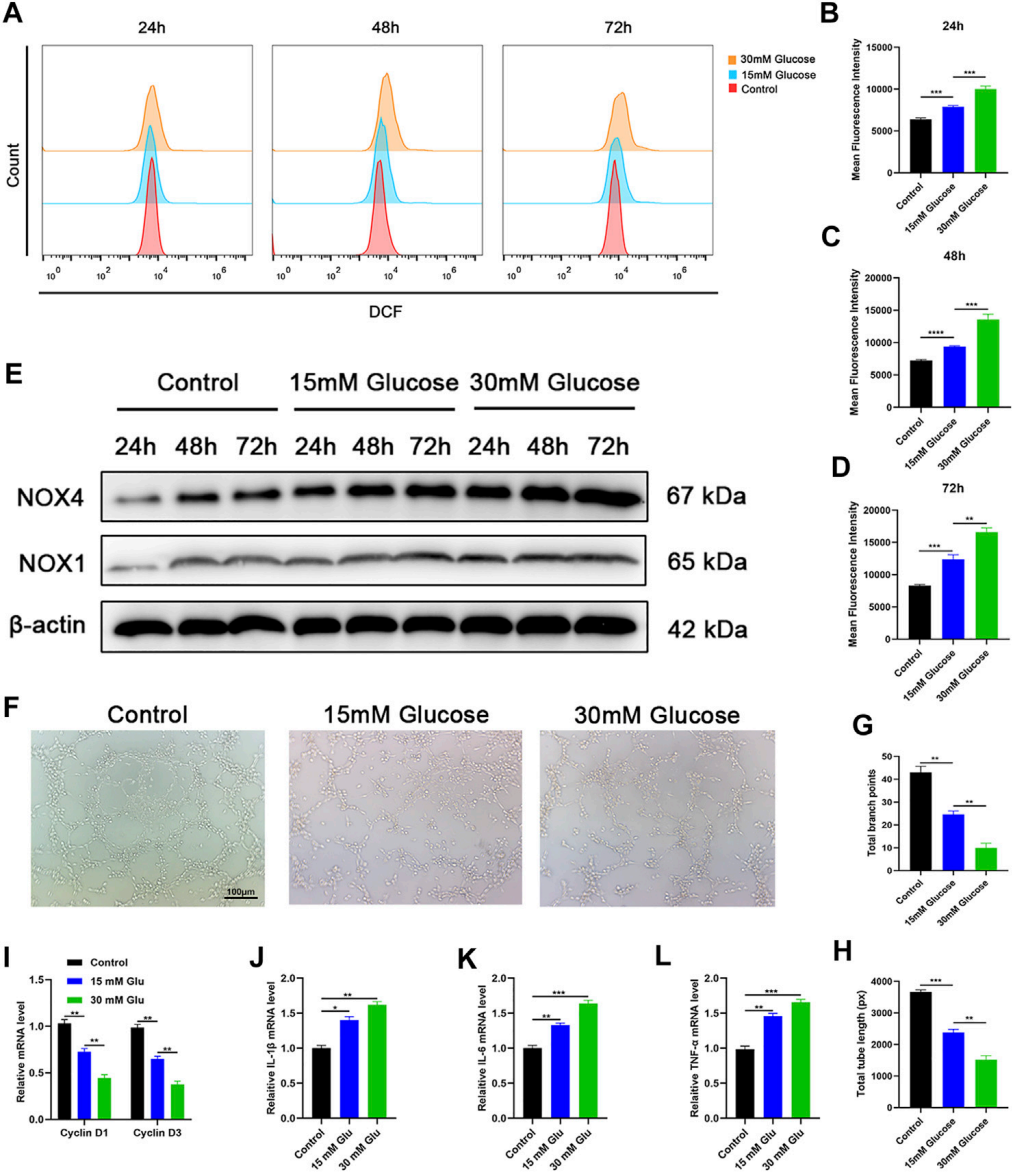
Hyperglycemia drives oxidative stress damage and impairs cellular function in HUVECs. **(A)** Reduction of reactive oxygen species (ROS) in HUVECs assessed *via* flow cytometry with the DCFH-DA (2′,7′-dichlorodihydrofluorescein diacetate) probe following different treatments. **(B–D)** Quantitation of ROS depletion measured by the intensity of fluorescence of HUVECs. **(E)** Western blotting results of NOX1 and NOX4 in HUVECs following different treatments at varied time points. **(F)** A tube formation assay was performed to visualize the cell capillary network formation of HUVECs. Scale bar: 100 μm. **(G, H)** Quantitative analysis of the tube formation of HUVECs in the three groups. **(I)** RT-qPCR results of cyclin D1 and cyclin D3 expressions in the HUVECs following different treatments; **(J–L)** RT-qPCR results of IL-1β, IL-6, and TNF-α expressions in the HUVECs following different treatments. Differences were measured by one-way ANOVA followed by a Tukey *post hoc* test for pairwise comparison. Data presented as means ± SD. ****p < 0.0001, ***p < 0.001, **p < 0.01, and *p < 0.05.

To evaluate the oxidative stress damage caused by the hyperglycemic environment, we set up three groups of HUVECs in a culture medium with different glucose concentrations. The glucose concentrations in the culture medium were the normal physiological level (physiological levels quoted in our article refer to glucose concentration in the medium itself) and supranormal level (15 and 30 mM), respectively. Then we measured the oxidative stress level of each group at three time points: 24, 48, and 72 h after the incubation. Based on a previous study, DCFH-DA (2′,7′-dichlorodihydrofluorescein diacetate), as a ROS indicator, has the ability to visualize intracellular ROS variations (Jang and Sharkis, 2007). ROS flow fluorescence assay was performed in each group at the designated time points. Results show that the cells treated with high-level glucose concentrations had higher fluorescence intensity compared with the low-level group and middle-level group. Under the same conditions, the fluorescence intensity increased with the increase of culture time (Figure 1A). The same result is illustrated in the fluorescence intensity statistics (Figures 1B–D). Thus, the results showed that a high-glucose environment indeed induces oxidative stress in cells, and this effect increases with the duration of hyperglycemia. As mentioned above, NOX1 and NOX4, as oxidative stress-related factors, play an important role in the oxidative stress of endothelial cells. Besides, we detected the expression levels of oxidative stress-related factors at different time points in each group through Western blotting, and its outcome confirms that the expression levels of oxidative stress-related factors increased with time (Figure 1E). With the intention to detect endothelial cell activity, the tube formation experiment was performed, and the results showed that tube formation is reduced in a high-glucose environment (Figure 1F). The statistics of the tube formation experiment show the same result (Figures 1G, H). Cell proliferation-related factors cyclin D1 and cyclin D3 were analyzed by RT-qPCR, and the results of each group were consistent with Cell Counting Kit-8 (CCK-8) (Figure 1I). In addition, we evaluated the level of cell damage on HUVECs as reflected by the inflammatory response. The inflammatory factors IL-1β, IL-6, and TNF-α were analyzed by RT-qPCR, and the results showed that the intensity of the inflammatory response increased with the increase of glucose concentration (Figures 1J–L). The proliferation ability of HUVECs in each group was detected by Cell Counting Kit-8 (CCK-8) technology, and the results showed that the proliferation level of cells in the high-glucose group decreased compared with other groups, and this result became more significant over time (Supplementary Figure S1A). Together, these findings reflected that hyperglycemia drives oxidative stress damage and impairs cellular function in HUVECs.

### HUCMSCs can regulate oxidative stress damage of HUVECs through exosomes

**FIGURE 2:**
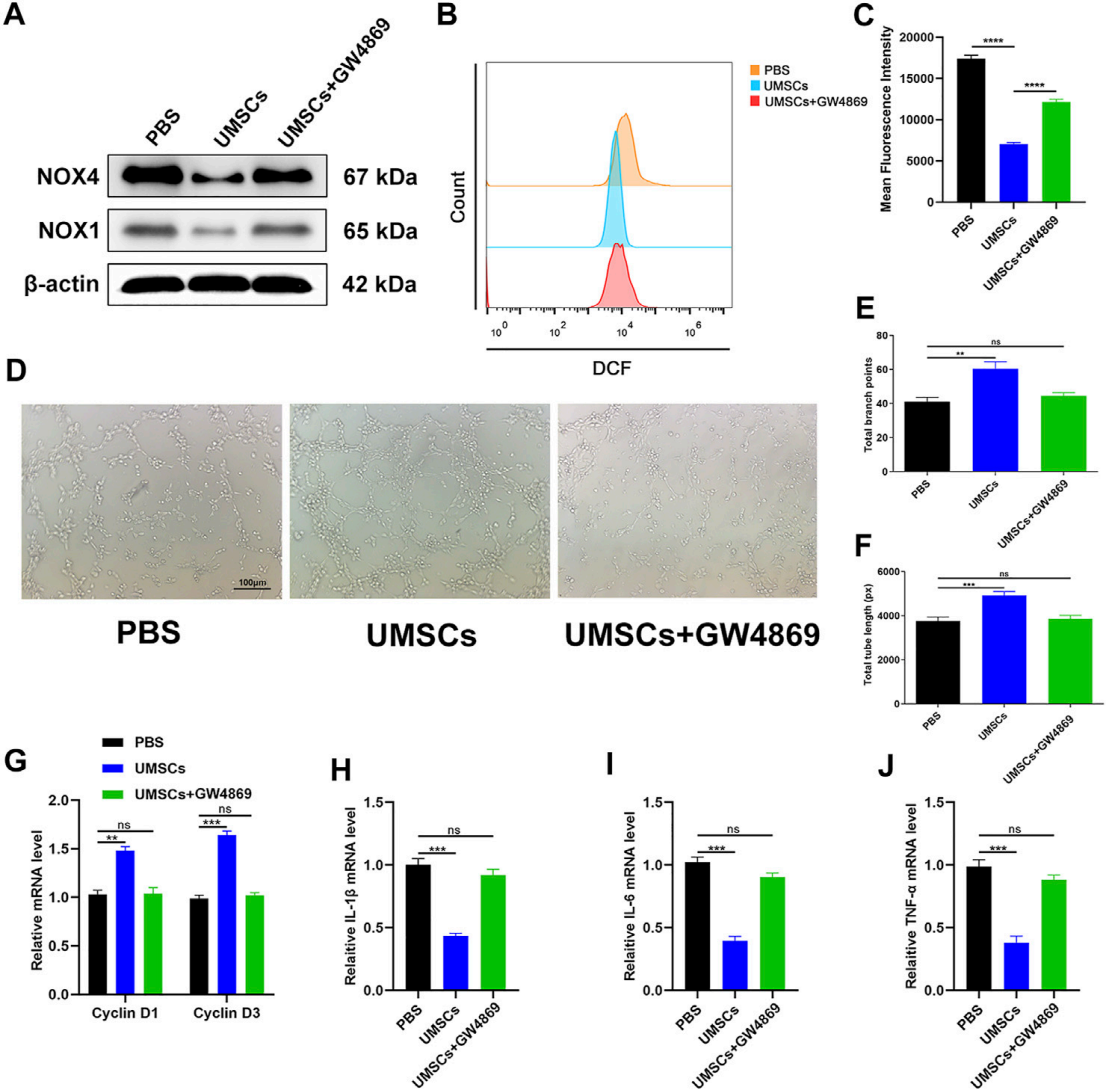
HUCMSCs can regulate oxidative stress damage of HUVECs through exosomes. **(A)** Western blotting results of NOX1 and NOX4 in HUVECs following different treatments. **(B)** Reduction of ROS in HUVECs assessed through flow cytometry with the DCFH-DA probe following different treatments. **(C)** Quantitation of ROS depletion measured by the intensity of fluorescence of HUVECs. **(D)** A tube formation assay was performed to visualize the cell capillary network formation of HUVECs. Scale bar: 100 μm. **(E, F)** Quantitative analysis of the tube formation of HUVECs in the three groups. **(G)** RT-qPCR results of cyclin D1 and cyclin D3 expressions in the HUVECs following different treatments. **(H–J)** RT-qPCR results of IL-1β, IL-6, and TNF-α expressions in the HUVECs following different treatments. Differences were measured by one-way ANOVA followed by a Tukey *post hoc* test for pairwise comparison. Data presented as means ± SD. ****p < 0.0001, ***p < 0.001, **p < 0.01, and *p < 0.05.

The effect of HUCMSCs on the regulation of oxidative stress damage in HUVECs was assessed. According to a previous study, GW4869 (hydrochloride hydrate) was utilized as an exosome secretion inhibitor (Dinkins et al., 2014). We established three groups of growth environments for HUVECs. The first group was HUVECs incubated in a 30-mM glucose medium. The second group was a coincubated system consisting of HUCMSCs and HUVECs incubated in a 30-mM glucose medium. Based on the condition of the second group, GW4869 was added into the third group to inhibit the secretion of exosomes. Based on the outcome shown in Figure 1E, we evaluated the expression levels of NOX1 and NOX4 in each group at the time point of 72 h through Western blotting. The results revealed that the first and third groups express increased levels of oxidative stress-related factors, while the second group showed the opposite consequence (Figure 2A). ROS flow fluorescence assay demonstrated that HUCMSCs reduced the fluorescence in the second group compared with the others. The third group, on the other hand, demonstrated a strong fluorescence. The statistical significance was detected between the groups (Figures 2B, C). Tube formation was performed to evaluate the capillary network formation by HUVECs. As shown in the results, the endothelial cells of the second group showed higher tube-forming ability, while the cells in the third group were hardly better than the first group in tube formation (Figures 2D–F). Then we conducted RT-qPCR, as a supplement, to analyze the expression levels of cyclin D1 and cyclin D3. The results showed that proliferation-related factors in the second group had higher levels than the other groups (Figure 2G). In order to evaluate the inflammatory response, we then employed RT-qPCR to detect IL-1β, IL-6, and TNF-α as surrogate markers of cell damage. The results showed that the levels of inflammatory factors in the second group were lower than that of the other groups, and the third group had no significant difference from the first group (Figures 2H–J). A CCK-8 experiment was conducted, and the outcome reflected that the cell proliferation ability of the second group was significantly better than the other groups, and the third group was slightly higher than the first group (Supplementary Figure S1B). These data indicated that HUCMSCs improve oxidative stress damage of HUVECs through exosomes derived from HUCMSCs.

### Identification of exosomes secreted by HUCMSCs

**FIGURE 3:**
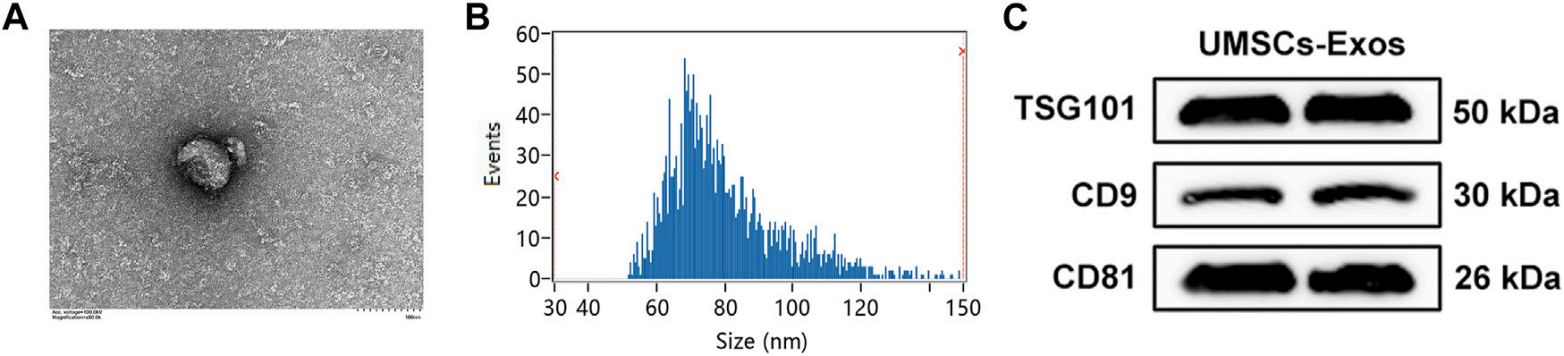
Identification of HUC-Exos (exosomes secreted by HUCMSCs). **(A)** Representative picture of the ultrastructure of exosomes observed by transmission electron microscopy (TEM). **(B)** The average particle size distribution of normal-Exos and diabetes-Exos was measured by dynamic light scattering (DLS). **(C)** The marker protein levels of CD9, CD81, and TSG101 in the isolated exosomes were detected with Western blotting. Differences were measured by one-way ANOVA followed by a Tukey *post hoc* test for pairwise comparison. Data presented as means ± SD. ****p < 0.0001, ***p < 0.001, **p < 0.01, and *p < 0.05.

To investigate the effect of HUC-Exos (exosomes secreted by HUCMSCs) on diabetic cutaneous wound healing, we assessed HUC-Exos through transmission electron microscopy (TEM), dynamic light scattering (DLS), and Western blotting. We began by collecting HUCMSCs to isolate exosomes. Analyzing the TEM data, we found that HUC-Exos included typical structures of exosomes, known to be homogeneous, spherical, and membrane vesicles (Figure 3A). The TEM images of HUC-Exos are also in agreement with a previous study. DLS was conducted to verify the particle size of HUC-Exos and to further identify the characteristic of HUC-Exos. According to the DLS data, particle sizes of HUC-Exos ranged from 30 to 150 nm (Figure 3B). Western blotting demonstrated that these isolated particles had high expression levels of CD9, CD81, and tumor susceptibility gene 101 (TSG101), which are typical markers of exosomes (Figure 3C). Together, these results revealed that the nanoparticles isolated from HUCMSCs were exosomes.

### Exosomes secreted by HUCMSCs are the mechanism underlying the beneficial effect of HUCMSCs on HUVECs

**FIGURE 4:**
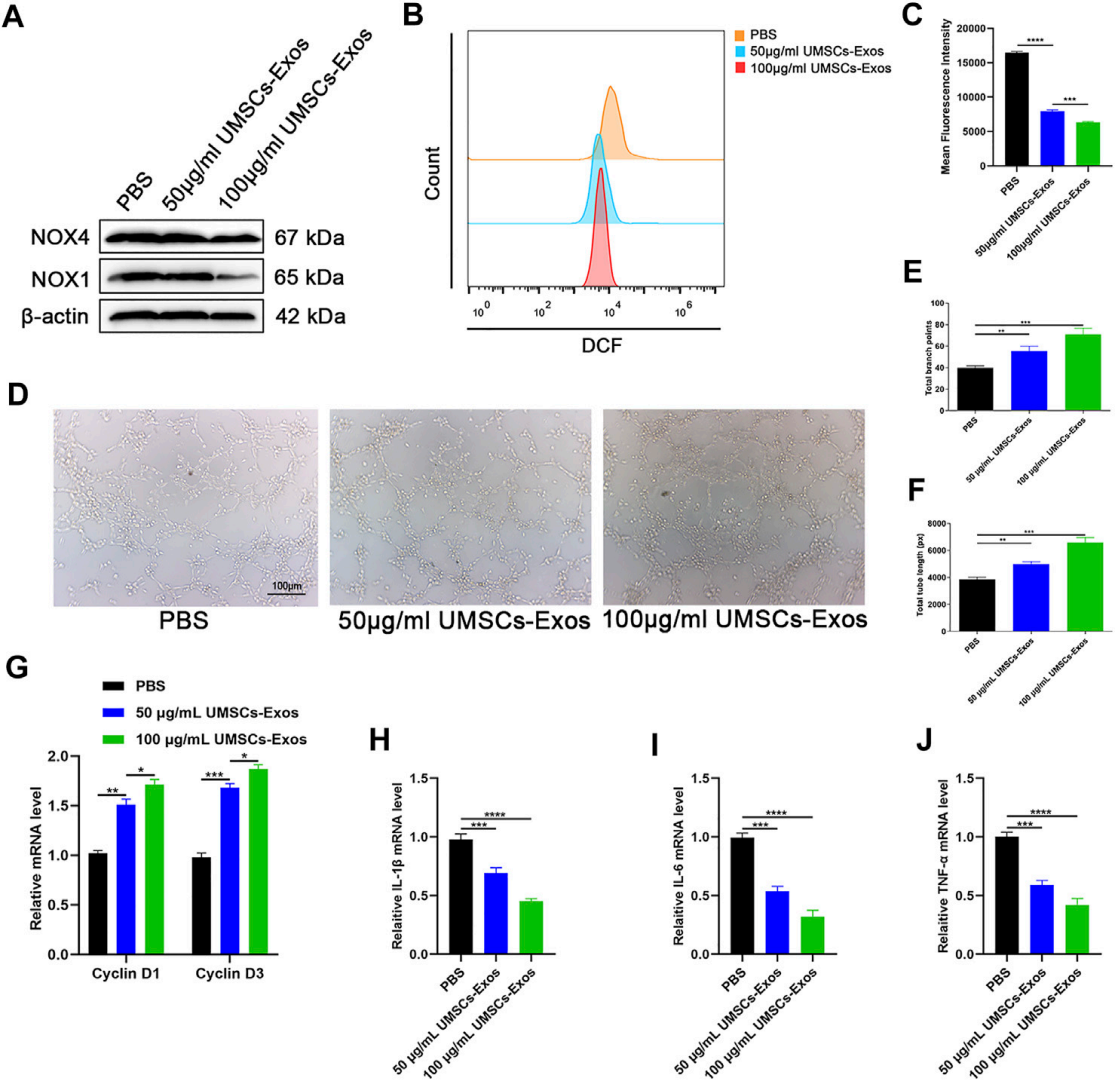
HUC-Exos can improve oxidative stress injury caused by hyperglycemia *in vitro*. **(A)** Western blotting results of NOX1 and NOX4 in HUVECs following different treatments. **(B)** Reduction of ROS in HUVECs assessed by flow cytometry with the DCFH-DA probe following different treatments. **(C)** Quantitation of ROS depletion measured by the intensity of fluorescence of HUVECs. **(D)** A tube formation assay was performed to visualize the cell capillary network formation of HUVECs. Scale bar: 100 μm. **(E, F)** A tube formation assay was performed to visualize the cell capillary network formation of HUVECs. Scale bar: 100 μm. **(G)** RT-qPCR results of cyclin D1 and cyclin D3 expressions in the HUVECs following different treatments. **(H–J)** RT-qPCR results of IL-1β, IL-6, and TNF-α expressions in the HUVECs following different treatments. Differences were measured by one-way ANOVA followed by a Tukey *post hoc* test for pairwise comparison. Data presented as means ± SD. ****p < 0.0001, ***p < 0.001, **p < 0.01, and *p < 0.05.

To determine the effect of HUC-Exos in improving oxidative stress injury, three groups were founded based on the medium. The common feature of the three groups was that HUVECs were cultured in the medium with a glucose concentration of 30 mM. The three groups were successively supplemented with 0, 50, and 100 μg/ml of HUC-Exos. Then according to the results above, we selected the time point of 72 h to evaluate the expression levels of NOX1 and NOX4 in HUVECs through Western blotting. It was shown that oxidative stress factors were expressed at lower levels in higher concentrations of exosomes (Figure 4A). ROS flow fluorescence assay was conducted, and the results showed that fluorescence intensity decreased with the increase in the exosome concentrations, as highlighted by the results of the statistical analyses (Figures 4B, C). With the intention to verify the function of HUVECs, we performed a tube formation experiment. The results showed that endothelial cell tubule formation in the high-concentration exosome group was higher than that in the low-concentration group (Figures 4D–F). In addition, endothelial cell proliferation was detected *via* RT-qPCR, and data showed that the expression levels of cyclin D1 and cyclin D3 increased with the elevation of the HUC-Exos concentrations in the culture medium (Figure 4G). Further, RT-qPCR was performed to detect the expression of endothelial inflammatory factors in a high-glucose environment. We found that the expression levels of IL-1β, IL-6, and TNF-α were negatively correlated with the concentration of exosomes in the medium (Figures 4H–J). CCK-8 was performed to further identify the proliferation ability of endothelial cells, and the data reflected that endothelial cell proliferation was positively correlated with exosome concentration (Supplementary Figure S1C). Based on these data above, it can be concluded that HUC-Exos improve oxidative stress injury caused by hyperglycemia *in vitro*.

### Exosomes secreted by HUCMSCs accelerate diabetic cutaneous wound healing and enhance angiogenesis *in vivo*

**FIGURE 5:**
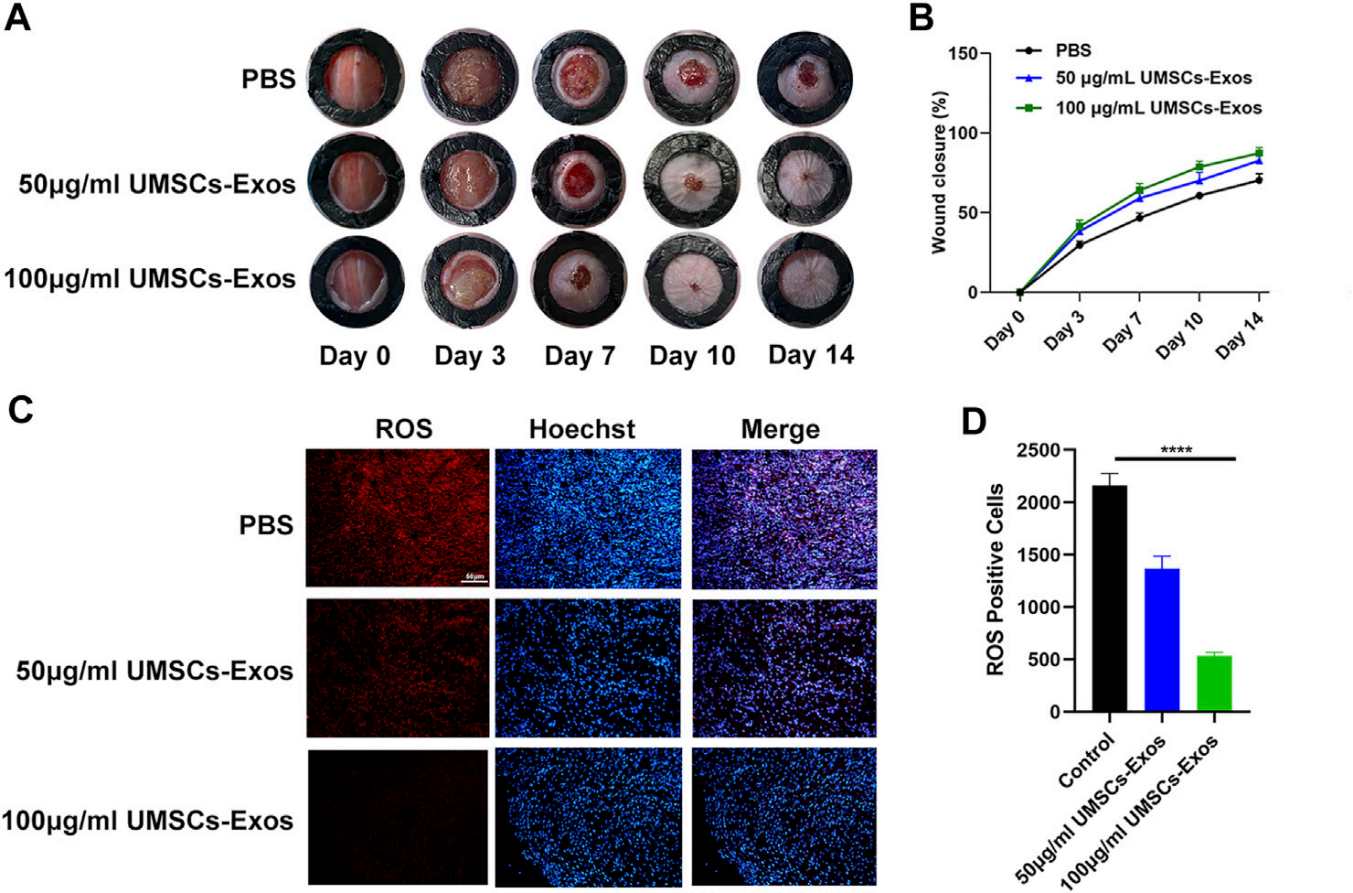
HUC-Exos accelerate diabetic cutaneous wound healing *in vivo*. **(A)** Representative images of the wound healing process of mice treated with PBS, 50 μg/ml of UMSCs-Exos, and 100 μg/ml of UMSCs-Exos (n = 10). **(B)** *In vivo* wound closure rates of the five groups at different time points. **(C)** ROS level assessed by immunohistochemistry (IHC) staining. **(D)** Fluorescence quantification of the wound length at day 14. Scale bar: 50 μm. Differences were measured by one-way ANOVA followed by a Tukey *post hoc* test for pairwise comparison. Data presented as means ± SD. ****p < 0.0001, ***p < 0.001, **p < 0.01, and *p < 0.05.

**FIGURE 6:**
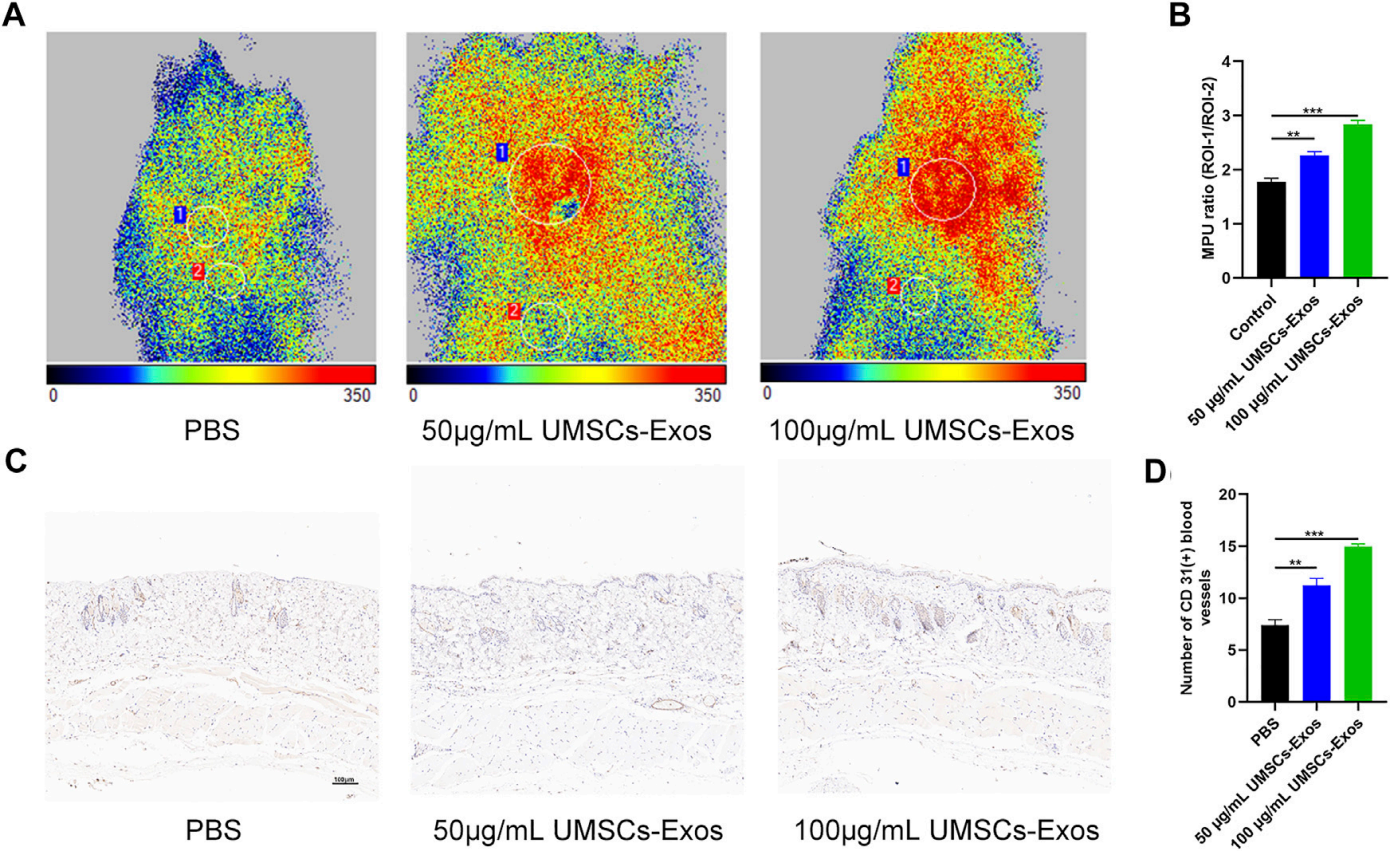
HUC-Exos enhanced angiogenesis *in vivo*. Mice were treated with PBS, 50 μg/ml of UMSCs-Exos, and 100 μg/ml of UMSCs-Exos (n = 10). **(A)** The perfusion of the wound area in the three groups was assessed using a small-animal Doppler analysis. Scale bar: 100 μm. **(B)** Quantitative perfusion intensity of the wound area in the three groups. **(C)** Immunofluorescence assay with marker CD31 (+), n = 10. Scale bar: 100 μm. **(D)** Fluorescence quantification of the wound length at day 14. Scale bar: 50 μm. Differences were measured by one-way ANOVA followed by a Tukey *post hoc* test for pairwise comparison. Data presented as means ± SD. ****p < 0.0001, ***p < 0.001, **p < 0.01, and *p < 0.05.

To characterize the effect HUC-Exos exerted on accelerating diabetic cutaneous wound healing, a mouse model of diabetic cutaneous wounds was produced, and the effects of HUC-Exos were studied. Three groups were established, and each group was given an equal amount of PBS, 50 μg/ml of UMSCs-Exos, and 100 μg/ml of UMSCs-Exos to the wound site through local injections. As shown in Figures 5A, B, the wound healing of the high-concentration exosome group was fastest among the groups, followed by the low-concentration exosome group. So the healing curve is shown (Figures 5A, B). In addition, we collected the tissue around the wound site from each group 14 days after induction of the wounds. Dihydroethidium (DHE) staining was carried out to evaluate the ROS content. Wounds treated with HUC-Exos showed a better granulation tissue formation than the other groups, and the group treated with the high concentration of exosomes showed the highest level of tissue formation (Figure 5C). The fluorescence intensity statistics also reflect the same results (Figure 5D). To evaluate whether angiogenesis was regulated by HUC-Exos, we collected the tissue around the wound site from each group 10 days after induction of the wounds for small-animal Doppler examination. The data demonstrated that the high-concentration exosome group had better blood perfusion as reflected by the mean perfusion unit (MPU) ratio, which is consistent with the result expressed by the Doppler intensity graph (Figures 6A, B). Further, immunohistochemistry (IHC) staining was applied to wound tissue samples 14 days after induction of the wounds, and the CD31 (+) cells were used as the main indicator of angiogenesis. As shown in the results, the density of CD31 (+) cells was significantly higher in the group with a high level of HUC-Exos, indicating greater angiogenesis (Figure 6C). Immunofluorescence intensity statistics also reflect the same results (Figure 6D). Taking all these data into consideration, we found that HUC-Exos do accelerate diabetic cutaneous wound healing *in vivo*.

## Discussion

In this study, we found that HUCMSCs significantly inhibited oxidative stress damage induced by high glucose in HUVECs, thereby regulating their activity, proliferation, and angiogenesis. HUCMSCs enter HUVECs by secreting exosomes through endocytosis. HUC-Exos regulate endothelial cell activity and function by inhibiting oxidative stress and reducing inflammatory response, thereby promoting angiogenesis at the macrolevel. In addition, we found that HUC-Exos promote wound healing primarily by regulating reepithelialization, collagen deposition, and ECM remodeling. Combined with these results, it appears that HUC-Exos regulate endothelial cell function by reducing oxidative stress and inflammatory response, thereby promoting angiogenesis and ultimately accelerating diabetic wound healing. It offers a promising strategy for improving diabetes.

Diabetic cutaneous wounds are typified by drug-resistant bacterial infections, compromised angiogenesis, and oxidative damage to the microenvironment (Luo et al., 2021). The reconstruction of skin integrity and conservation of appropriate blood supply are the key factors for wound healing (Wong et al., 2015; Castleberry et al., 2016; Wang et al., 2019). According to previous studies, vascular complications caused by diabetes lead to endothelial dysfunction and hinder vascular repair (Zhang and Sun, 2020). More evidence indicates that the important cause of vascular dysfunction is endothelial cell function impairment induced by hyperglycemia, which leads to the occurrence of diabetic complications (Luo et al., 2020). Mechanistically, high glucose can induce endothelial cell apoptosis and dysfunction by activating the NF-κB signaling pathway, thereby inhibiting angiogenesis at the tissue level (Lee et al., 2019). In addition, a previous study reported that a high-glucose environment can inhibit endothelial cell activity, induce cell apoptosis, and induce oxidative stress injuries of HUVECs, making diabetic patients vulnerable to vascular diseases (Liu et al., 2020). In this study, our data also supported the above conclusion that a high-glucose environment caused oxidative stress damage and inflammatory response in endothelial cells and inhibited the functional activity and proliferation ability of endothelial cells (Figure 1).

HUCMSCs are pluripotent stem cells with omnidirectional differentiation potentials, which can regulate the functional and repair ability of various tissues and cells (Toh et al., 2017; Philipp et al., 2018). Our study showed that HUCMSCs regulate the functional activity and proliferation of endothelial cells (Figure 2). In addition, according to previous reports, HUCMSCs play a therapeutic role in tissue repair, mainly through paracrine exosomes (Corley et al., 2017; Wang et al., 2021). Exosomes are small vesicles secreted into the circulation by a series of cell types in the body and internalized by proximal or distal cells. These small molecules (including proteins and nucleic acids) in exosomes regulate the function of the recipient cells after internalization (Yang et al., 2019; Isaac et al., 2021). The outside secrete body has proven to be an ideal nanomaterial because it protects its contents, such as microRNAs, lncRNAs, and proteins. From the interference of external factors, the material is passed to the target cell, thus regulating gene expression and function in the target cell, allowing paracrine and endocrine communications between different tissues (Liu and Su, 2019). Exosome-based cell-free therapy avoids side effects associated with cell therapy, such as immune rejection and ectopic tissue formation (Mi et al., 2020; Wang et al., 2021). Our results showed that HUC-Exos entered endothelial cells through endocytosis and had the effect of inhibiting inflammatory response and alleviating oxidative stress (Figure 4). *In vitro* experiments also supported the above conclusions (Figure 5).

There is no doubt that the specific mechanism of HUC-Exos on endothelial cells in our study needs to be further studied. According to recent reports, the proliferation and tubulogenesis of vascular endothelial cells transfected with miR-20b-5p from peripheral blood exosomes in diabetic patients were significantly reduced, and the apoptosis rate was significantly increased (Niemiec et al., 2020). In addition, exosome-derived miR-146a significantly decreased phosphorylated IκB-α and NF-κB, thereby regulating the function of vascular endothelial cells (Xiong et al., 2020). From a therapeutic perspective, previous studies have pointed out that the self-levitating nanofiber gel-encapsulated polydeoxyribonucleotide benefits chronic ulcer in diabetic rats (Chen et al., 2016). The research of Xiong Y et al. indicates that chronic diabetic wound repair benefits from the nanohydrogel material loaded with growth factors (Xiong et al., 2021). At present, clinical trials have confirmed that miRNA is a key regulatory molecule of fracture healing and is likely to be a pathway to promote normal physiologic fracture healing. Its easy combination with agonists or antagonists makes it an ideal target for the treatment of fracture nonunion (Komatsu et al., 2021). All these provide possible directions for further research on the mechanism. In this study, human umbilical vein mesenchymal stem cells modulated endothelial cell injury and promoted fracture healing by secreting exosomes. However, the regulation from secretory exosomes to endothelial cells has not been further discussed. Although possible modes of action and potential targets were listed in the discussion section, they were not reflected in the experiment. This will be the direction of our further research in the future.

## Conclusion

Taken together, our results show that HUC-Exos accelerate diabetic cutaneous wound healing *via* ameliorating oxidative stress and enhancing angiogenesis. From the point of view of clinical utility, this research could lead to addressing diabetic cutaneous wounds in the form of one-step mixed injection, thus reducing the medical burden and simultaneously increasing economic benefits. To summarize, our research provides a promising therapeutic approach to promote diabetic wound healing in the future.

## Methods and materials

### Cell culture and transfection

Experiments enrolled in this study were authorized by the Ethics Committee, Tongji Medical College, Huazhong University of Science and Technology, China.

HUVECs were offered by the Cell Bank of the Chinese Academy of Science, Shanghai, China. The cell culture medium is RPMI 1640 (Sigma-Aldrich, USA, cat. no. R8758), containing 10% exosome-depleted FBS (BI Israel).

For HUCMSCs, fresh umbilical cords were derived from informed pregnant women at the Wuhan Union Hospital (China). PBS was utilized to rinse the umbilical cords twice, and 5% penicillin-and-streptomycin PBS was used to rinse the umbilical cords three times.

Researchers removed cord vessels, and the processed cords were subsequently divided into small pieces that were individually attached to the substrate of culture plates. The stem cell culture medium (Beyotime, China, cat. no. S0154s) was hired, and the cells were incubated at 37°C with 5% CO_2_. Then the cells changed the medium every 3 days.

And 10 days after the first incubation, the cells were well-developed and ready for experiments.

Inclusion criteria for pregnant women are as follows: 1) adult females aged 22–28; 2) the delivery condition was good, and the BPS score was more than 8; 3) pregnant women have no other adverse diseases; and 4) pregnant women who signed informed consent.

Exclusion criteria for pregnant women are as follows: 1) pregnant women with twins or more at the same time, 2) pregnant women with severe obstetric complications, and 3) pregnant women with poor prognosis after secretion.

HUCMSCs were grown in the RPMI 1640 medium (Sigma-Aldrich, USA, cat. no. R8758) supplemented with 10% fetal bovine serum (Gibco) and 1% pen/strep (Gibco). The cells were incubated in 5% CO_2_ at 37°C.

### Exosome purification and characterization

HUCMSCs were filtered through a 0.2-µm filter (122-0020PK, Thermo Fisher Scientific) at 4°C. The pellet was washed in PBS, resuspended, and centrifuged for further 20 min at 100,000 × *g*, and the exosome-containing pellet was resuspended in PBS. Transmission electron microscopy (TEM, Tecnai G2) was used to evaluate the morphology of Exos. A Nanosizer instrument (Malvern Instruments, Malvern, UK) was used for dynamic light scattering analysis, and Western blotting was performed to analyze exosomal surface markers. The expressions of surface markers were determined by flow cytometry.

### Tube formation assay

Cells (2 × 10^5^ per well) were incubated in 24-well plates and grown for 1 day with mentioned treatment. HUVECs (2 × 10^4^/well) were plated in 96-well plates precoated with Matrigel, incubated for 45 min, and then incubated for further 8 h. Three randomly chosen fields were examined using an inverted microscope, and the branch points and tube lengths were measured using ImageJ. We use gray analysis and other quantitative functions to transform pictures and tables.

### GW4869

Product specification is cat. no. 52321ES10, 10 mg, YEASEN Biotech Co., Ltd., China. A 1.5-mm storage solution was prepared with DMSO and stored at −80°C. Before use, 5% MSA was dissolved into GW4869 storage solution, making GW4869 1.43 mM. The suspension is thoroughly mixed and heated at 37°C until clear. Then the GW4869 inhibitor was added to the cell culture medium at a concentration range of 10–20 μM, and the cells were treated for 30 min.

### Western blotting analysis

The proteins of HUVECs were isolated using a RIPA lysis solution (Gibco, China). After treatment, HUVECs were rinsed with cold PBS twice, and RIPA lysis solution was added to the cells. The protein extracts were harvested at 12,000 *g* at 4°C for 10 min. Protein concentration was detected using a BCA protein assay kit (Beyotime, China). Equal amounts of protein were separated by 10%–12% sodium dodecyl sulfate–polyacrylamide gel electrophoresis and transferred to PVDF membranes (MilliporeSigma, USA). The membranes were blocked with 5% nonfat milk and incubated with the primary antibodies overnight at 4°C. The membranes were rinsed with PBS-T twice and incubated with the appropriate secondary antibodies at room temperature for 1 h. Subsequently, the proteins of the membranes were visualized according to the instructions of the manufacturer. The following antibodies were used: anti-TSG101 (1:1,000; Abcam, cat. no. Ab125011), anti-CD9 (1:1,000; Abcam, cat. no. Ab92726), anti-CD81 (1:1,000; Abcam, cat. no. Ab82452), anti-NOX1 (OCN; 1:500; Abcam, cat. no. Ab153241), anti-NOX4 (OCN; 1:500; Abcam, cat. no. Ab198213), and anti-GAPDH (1:10,000; Abcam, cat. no. Ab37168). All experiments were performed in triplicate.

### RT-qPCR analysis

TRIzol (Invitrogen) was utilized to extract total RNA, and a Verso cDNA Synthesis Kit (Thermo Fisher Scientific) was used to reverse-transcribe the RNA according to the instructions of the kit. The SeraMir Exosome RNA Purification Kit (System Biosciences, Mountain View, USA) was used to extract exosomal miRNAs, and the TaqMan microRNA assay kit (Applied Biosystems, Foster City, USA) was utilized for cDNA synthesis. The StepOne Real-Time PCR System (Life Technologies, Carlsbad, CA, USA) was used for RT-qPCR reactions. GAPDH and U6 were used for the normalization of mRNA and miRNA expressions, respectively, and the 2^−ΔΔCt^ method was used to quantify relative expressions: IL-1β (Abcam, UK), IL-6 (Abcam, UK), TNF-α (Abcam, UK), cyclin D1 (Abcam, UK), and cyclin D3 (Abcam, UK).

### Reactive oxygen species assay

The ROS Assay Kit (Beyotime, China, cat. no. S033SS), containing DCFH-DA (the fluorescent probe) and ROSup (ROS positive control), was stored at −20°C. After the cells were removed from the culture medium, 1 ml of 10 mm/L DCFH-DA was added. The cells were then incubated in an incubator at 37°C for 20 min. The cells were then washed three times in a serum-free culture medium. For flow cytometry detection, the excitation wavelength was 488nm and the emission wavelength was 525 nm.

### Generation of diabetic mice

The diabetes model was established in male C57BL/6J mice (6 weeks old) by feeding on a high-fat diet for 4 weeks, followed by daily intraperitoneal administration of streptozotocin (STZ; 40 mg kg^−1^ day^−1^) for 7 days. If the fasting blood glucose level was over 11.1 mmol L^−1^ for two successive measurements, the mice were considered to be diabetic and were used for the following experiments.

### Murine wound model

The STZ-induced diabetic mice were anesthetized with intraperitoneal pentobarbital sodium (50 mg kg^−1^; Sigma-Aldrich), and 1.0 cm × 1.0 cm full-thickness excision skin wounds were made. The mice were randomly divided into groups and injected with 100 µl of PBS, 50 μg/ml of UMSCs-Exos, and 100 μg/ml of UMSCs-Exos on days 0, 3, 5, 7, 9, and 11 after the establishment of the wound (n = 6). The wounds were covered with transparent dressings (Tegaderm™ Film) and were photographed and measured with a caliper on days 0, 3, 5, 7, 10, and 14. The degree of wound closure was determined by ImageJ as follows:

$$C_{n}=\frac{\left(A_{0}-A_{n}\right)}{A_{0}}\times 100\%$$

where C_n_ is the percentage reduction of the wound area on the respective days; A_0_ is the size of the original wound; A_n_ is the area of the wound on the respective days after the injury.

### Dihydroethidium staining

Half of the wound tissue samples were collected on days 3, 7, and 14, fixed (4% formaldehyde), and paraffin-embedded. Sections (5 µm) were used in the DHE assay. Wound samples, including full-thickness skin layers, were cryosectioned. DHE staining (5 µm) was used to measure intracellular ROS. Images were acquired using the IX53 microscope (DHE kit; Abcam, cat. no. ab236206).

### Small-animal Doppler analysis

Ten days after the operation, a laser speckle contrast imaging (LSCI) system was utilized to examine local blood perfusion, while the PSI-ZR PeriCam system (Perimed Ltd., Stockholm, Sweden) was used to acquire images of the wounds. An invisible near-infrared (NIR) laser at 785 nm was used to determine blood perfusion, expressed as perfusion units. The wounds were photographed at a constant distance, using the same dimensions for the area. The MPU ratio was determined using PIMSoft (Moor Instruments Ltd., Axminster, UK) using flux images of individual wound sites, expressed as the correlation of the MPU of the wound area (ROI-1) to the MPU of the region surrounding the wound (ROI-2). The statistical map of fluorescence intensity was converted by FlowJ software.

### Immunohistochemistry staining

The mice were sacrificed on day 14, and the wound tissues were embedded in paraffin and stained for CD31. Antigen retrieval was performed for 1 min in citrate buffer, followed by blocking for 30 min in goat serum. The samples were stained with anti-CD31 (1:100; Abcam, ab28364) antibodies overnight at 4°C, washed in PBS, and stained and counterstained with DAB and hematoxylin, respectively. The sections were evaluated under a microscope (Nikon, Japan). The statistical map of fluorescence intensity was converted by FlowJ software.

### Origin of laboratory rats

Description of laboratory rats is as follows: variety: C57BL/6J; incubation company: Beijing Vital River, China; gender: 70 males and 70 females; age: 42 days; level: SPF; and license number: SCXK 2021-0010.

### Cell coculture system

A Transwell plate was used (Corning, China, the no.: 3,470, 1 µm). 1. For the preparation of the cell suspension, the cells were digested, the culture medium was discarded by centrifugation after the termination of digestion, washed once or twice with PBS, and the cell density was adjusted by resuspension with a serum-free medium containing BSA. 2. For cultured cells, Transwell was used to inoculate human umbilical cord mesenchymal stem cells and endothelial cells. 3. The morphological changes of the cells in the upper and lower chambers were observed under the microscope, and the staining identification could be carried out if necessary. 4. Results were statistically analyzed.

### Statistical analysis

The data were expressed as means ± standard deviation (SD). Differences between two groups were measured by Student’s t-tests, and differences between multiple groups were measured by one-way ANOVA followed by a Tukey *post hoc* test for pairwise comparison. Analyses were conducted with GraphPad Prism 9.0. *p* < 0.05 was set as the significance threshold.

## Data Availability

The original contributions presented in the study are publicly available. This data can be found here: https://doi.org/10.6084/m9.figshare.17696024.v1.
